# Antiviral Activity of PD-L1 and PD-L4, Type 1 Ribosome Inactivating Proteins from Leaves of *Phytolacca dioica* L. in the Pathosystem *Phaseolus vulgaris–*Tobacco Necrosis Virus (TNV)

**DOI:** 10.3390/toxins12080524

**Published:** 2020-08-14

**Authors:** Daniela Bulgari, Nicola Landi, Sara Ragucci, Franco Faoro, Antimo Di Maro

**Affiliations:** 1 Agri-food and Environmental Microbiology Platform (PiMiAA), Department of Molecular and Translational Medicine, University of Brescia, viale Europa, 11, 25123 Brescia, Italy; daniela.bulgari@unibs.it; 2 Department of Environmental, Biological and Pharmaceutical Sciences and Technologies (DiSTABiF), University of Campania ‘Luigi Vanvitelli’, Via Vivaldi 43, 81100 Caserta, Italy; nicola.landi@unicampania.it (N.L.); sara.ragucci@unicampania.it (S.R.); 3 Department of Agricultural and Environmental Sciences, University of Milan, Via Celoria 2, 20133 Milan, Italy; franco.faoro@unimi.it

**Keywords:** antiviral proteins, ribosome inactivating proteins, *Phytolacca dioica* L., protein purification, tobacco necrosis virus

## Abstract

Using the pathosystem *Phaseolus vulgaris*–tobacco necrosis virus (TNV), we demonstrated that PD-L1 and PD-L4, type-1 ribosome inactivating proteins (RIPs) from leaves of *Phytolacca dioica* L., possess a strong antiviral activity. This activity was exerted both when the RIPs and the virus were inoculated together in the same leaf and when they were inoculated or applied separately in the adaxial and abaxial leaf surfaces. This suggests that virus inhibition would mainly occur inside plant cells at the onset of infection. Histochemical studies showed that both PD-L1 and PD-L4 were not able to induce oxidative burst and cell death in treated leaves, which were instead elicited by inoculation of the virus alone. Furthermore, when RIPs and TNV were inoculated together, no sign of H_2_O_2_ deposits and cell death were detectable, indicating that the virus could have been inactivated in a very early stage of infection, before the elicitation of a hypersensitivity reaction. In conclusion, the strong antiviral activity is likely exerted inside host cells as soon the virus disassembles to start translation of the viral genome. This activity is likely directed towards both viral and ribosomal RNA, explaining the almost complete abolition of infection when virus and RIP enter together into the cells.

## 1. Introduction

Ribosome inactivating proteins (RIPs) are specific rRNA N-glycosylases present in various plants, fungi, and bacteria and potent inhibitors of protein synthesis [1]. Their mode of action is the specific depurination of major rRNA damaging ribosomes. In particular, these enzymes (EC: 3.2.2.22) cleave a specific adenosine (A_4324_, in the case of rat 28S rRNA) within a universally conserved region known as the Sarcin Ricin Loop (SRL) [2]. The irreversible cleavage of this single adenosine prevents the association between the elongation factors and ribosome, causing the inhibition of protein synthesis [3]. Furthermore, several authors report that RIPs are also able to remove adenine from different substrates such as polynucleotides, tRNAs and DNAs with a different grade of efficiency for which was proposed the name of adenine polynucleotide glycosylases (APGs; [4]) or are able to cleave phosphodiester bonds (DNase activity; [5,6]).

Structurally, RIPs are divided into two groups considering the presence or absence of a quaternary structure. Classically, these enzymes are categorized in monomeric RIPs (type 1) and dimeric RIPs (type 2). Type-1 RIPs consist in a single polypeptide chain with toxic N-glycosylase activity, while type 2 RIPs are constituted by a polypeptide chain exhibiting N-glycosylase activity (A-chain) linked to a lectin chain (B-chain) through a disulphide bond able to recognize carbohydrate (e.g., galactose/N-acetylgalactosamine) moieties of mammalian cell surface [7]. Alternatively, basing on the domain architecture and evolutionary background, type-1 and type-2 RIPs are called type-A and type-AB RIPs, respectively, while other chimeric forms as type-AX are grouped separately, where X indicates a different (unknown) domain found in the genomes of some Poaceae/cereal species [8,9].

Type 2 RIPs are more toxic in cellular systems (IC_50_ 0.0003–1.7 nM on Hela cell lines) with respect to type 1 (IC_50_ 170–3300 nM on Hela cell lines), while in acellular systems the toxicity of the two groups is comparable [10]. In addition, non-canonical RIPs, such as tetrameric RIPs from *Sambucus* [11] or proteolytic activated forms (pro-RIPs; i.e., maize b-32 [12]) were also found.

At the cellular level, the inhibition of protein synthesis by RIPs promotes cell death by apoptosis pathway considering that many studies report the activation of various caspases, caspase-like and serine proteases and poly(ADP-ribose) cleavage [13,14]. Moreover, the relationship between apoptosis and cell death often shows a difference in events succession due to the variation in intracellular routing of RIPs [15,16]. The ability of RIPs to kill target cells with and without specific carriers (e.g., antibodies, hormones, peptides, cytokines and protease inhibitors) is of great biomedical interest for the construction of specific “bullets” against cancer cells and in the treatment of viral or parasitic diseases [16,17,18,19].

RIPs have also been used in agriculture to protect crops from diseases caused by viruses and fungi, and from insect pests. Genes encoding for PAP, trichosanthin, and maize RIP are the most commonly used, while the main noted host plants are tobacco, potato, and tomato. Thus, transgenic plants carrying genes of some RIPs have been obtained with different degrees of resistance to viruses, fungi, and insects [20].

Despite the large number of works on RIPs field, the biological role of RIPs in plants has not been completely unveiled; although all researchers agree that RIPs are involved in plants defense or physiology [10,21]. Indeed, these toxins present antiviral properties, antifungal activities, defense role against antagonists or acting in plant processes such as programmed senescence, stress protection and regulation [20].

RIPs are found in a higher number of plants belonging to Caryophyllaceae, Sambucaceae, Cucurbitaceae, Euphorbiaceae, and Poaceae [22] that express various RIP isoforms encoded by multi-gene families [23]. In particular, a rich group of angiosperms expressing many type-1 RIPs is the Phytolaccaceae family [24], where the prototype of type-1 RIPs, pokeweed antiviral protein (PAP) from leaves of *Phytolacca americana* L., was isolated given its pronounced antiviral activity [25]. Since then, many others type-1 RIPs were isolated from Phytolaccaceae species such as *Phytolacca dodecandra* L’Herit [26], *Phytolacca heterotepala* H. Walter [27,28], *Phytolacca insularis* [29] Nakai, and *Phytolacca dioica* L. [30]. In particular, from seeds and from adult and young leaves of *P. dioica* plant, three (PD-S1-3; [31,32]), four (PD-L1-4; [33,34]), and two (dioicin 1 and 2; [35,36,37]) type-1 RIPs, respectively, were isolated and extensively characterized. On the other hand, biological and antipathogenic activities [6] and a possible source of antimicrobial peptides [38,39] have been recently reported for type-1 RIPs from *P. dioica*.

Despite the numerous type-1 RIPs isolated and studied from leaves of *P. dioica* very few information on their antiviral activity are available with respect to type-1 RIPs from *P. americana*. Therefore, in this work we report the antiviral activity of both PD-L1 and PD-L4, the major isoforms of type-1 RIPs expressed in leaves of *P. dioica* adult plant, by using the pathosystem tobacco necrosis virus (TNV)-*Phaseolus vulgaris* L. TNV is a member of the genus *Necrovirus* in the family *Tombusviridae* with unsegmented and uncapped TNV genome consisting of a single stranded linear positive sense RNA of 3.8 kb that lacks a poly A tail and replicates itself with the aid of its own RNA-dependent RNA polymerase [40]. In bean leaves, as well in many other plant species, TNV infection induces localized necrotic lesions due to the hypersensitive reaction (HR) elicited by the virus coat protein [40]. These lesions occur 3–4 days after infection and are easily quantifiable, thus this pathosystem is widely used to assess the level and mechanisms of induced plant resistance to viruses [41,42].

In this framework, the new information emerged by this work can be useful for deepening the knowledge on type-1 RIPs in *Phytolaccaceae* family.

## 2. Results and Discussion

### 2.1. Type-1 RIP Purification

**Figure 1:**
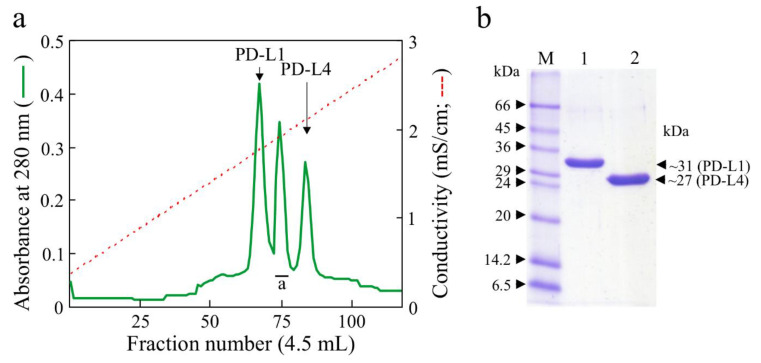
Purification of ribosome inactivating proteins (RIPs) from *Phytolacca dioica* leaves. (**a**) Elution profile from the CM-52 chromatography showing three main peaks. First and last peaks are PD-L1 and PD-L4, respectively. The second peak (peak a) was identified as PD-L2 and PD-L3 minority glycosylated isoforms of PD-L1 and PD-L4, respectively (Di Maro et al., 1999). (**b**) SDS-PAGE on 15% polyacrylamide under reducing conditions of the purified PD-L1 (lane 1) and PD-L4 (lane 2), respectively. M, molecular markers.

Native PD-L1 and PD-L4 were purified from fully expanded leaves of *P. dioica* as described previously using a general protocol for the preparation of basic proteins [33,35]. From a raw basic proteins extract from leaves, three chromatographic peaks were obtained from the last cation exchange chromatography step (Figure 1a). The first and the last peaks give homogeneous PD-L1 and PD-L4, respectively, while the second peak (peak a Figure 1a) contains simultaneously PD-L2 and PD-L3, glycosylated isoforms of PD-L1 and PD-L4 that were further not purified [34].

**Figure 2:**
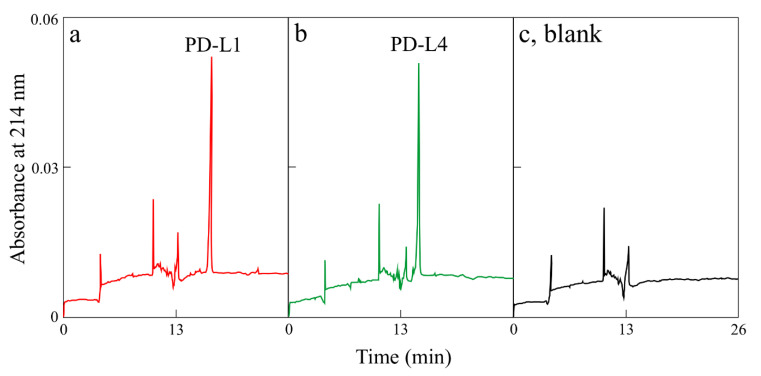
Capillary electrophoresis electropherograms of purified PD-L1 (**a**) and PD-L4 (**b**). In (**c**), reference electropherogram without proteins (blank).

Homogenous preparations of both PD-L1 and PD-L4, verified by SDS-PAGE (sodium dodecyl sulfate–polyacrylamide gel electrophoresis; Figure 1b) and capillary electrophoresis (Figure 2), were used for antiviral activity against TNV.

### 2.2. Antiviral Activity of PD-L1 and PD-L4

**Figure 3:**
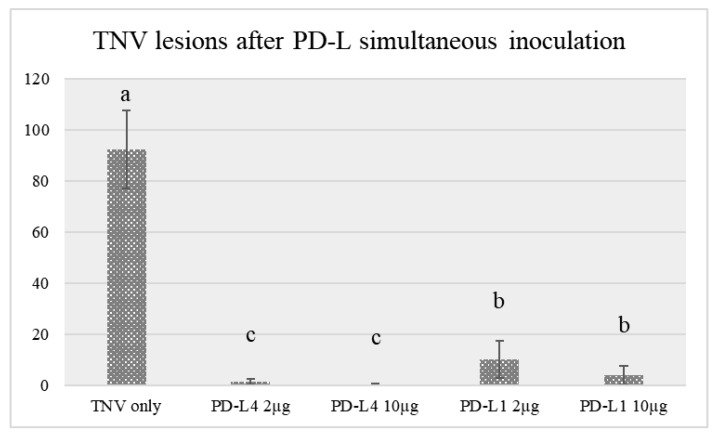
Number of tobacco necrosis virus (TNV) lesions developed after inoculation only with the virus or with the virus mixed to different concentrations of either PD-L1 or PD-L4. Different letters represent significant differences according to Fisher’s least significant difference test at *p* < 0.05. The error bars represent standard deviation.

Both PD-L1 and PD-L4 exerted a strong antiviral activity at the tested concentrations (2 and 10 µg/mL) when applied together with the virus suspension in the same bean leaf surface, as assessed by the reduction of lesion number with respect to bean plants inoculated only with the virus suspension in water (Table 1, Figure 3). However, PD-L4 antiviral activity was greater than PD-L1 and completely inhibited the appearance of visible virus lesions at a concentration of 10 μg/mL (−99.7%).

**Figure 4:**
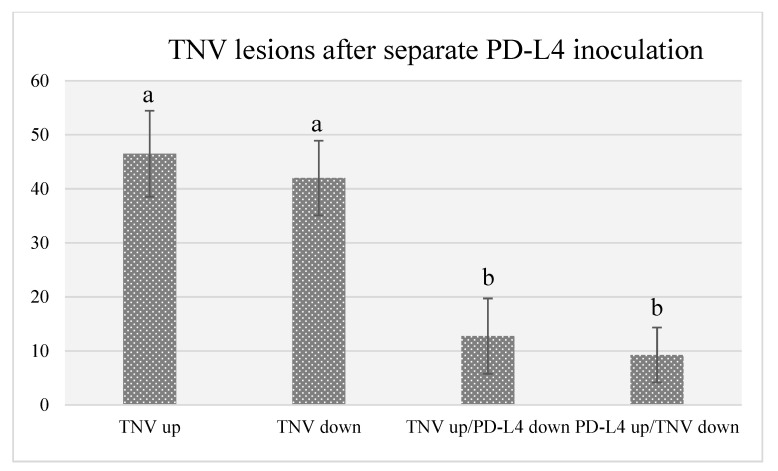
Number of TNV lesions developed when the virus was inoculated alone on the adaxial (up) or abaxial (down) leaf surface, or when it was inoculated separately from PD-L4 in the opposite leaf surface. Different letters represent significant differences according to Fisher’s least significant difference test at *p* < 0.05. The error bars represent standard deviation.

To verify if this inhibition was due to a direct effect of the RIPs on viral RNA and not to ribosomes inactivation, TNV was inoculated separately from RIPs. For this purpose, the virus suspension was rubbed either on the adaxial or abaxial leaf surface and PD-L4 on the opposite leaf surface. In these experiments, only PD-L4 was utilized being the most effective at the lowest concentration of 2 µg/mL. Results showed that the inhibitory activity of this RIP against TNV was partially weakened, but still high, with a reduction of lesion number over 70% with respect to controls (Table 1, Figure 4). Though we cannot exclude a direct contact of the virus with PD-L4, i.e., in the extracellular spaces, it is likely that this contact occurs directly in the first damaged cells following virus infection. Here, PD-L4 may exerts its activity both on replicating viral RNA and on ribosomal RNA. Indeed, in a previous paper it was demonstrated that PD-L4 is able to partially degrade tobacco mosaic virus RNA in vitro, thus supporting this hypothesis [6].

### 2.3. PD-L1 and PD-L4 Antiviral Activity is not Mediated by Cell Death and Oxidative Burst

**Figure 5:**
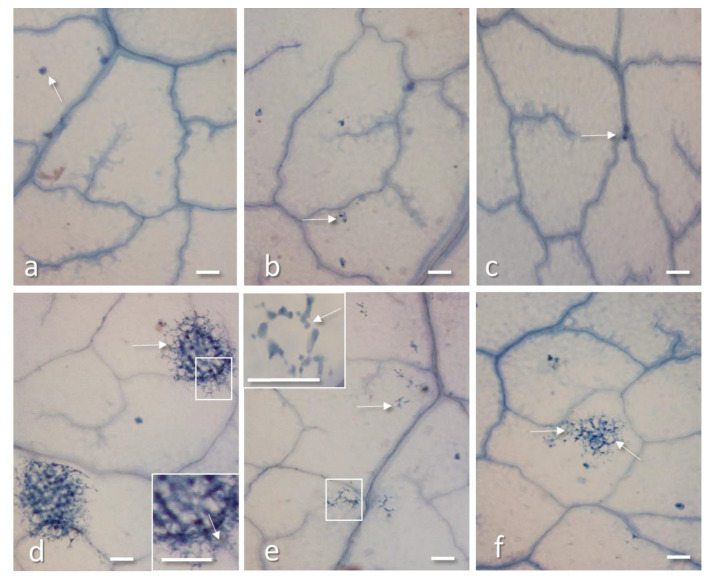
Evans blue staining of bean leaf disks 72 h after treatment with PD-L1 2 µg/mL (**a**) or PD-L4 2 µg/mL (**b**) or water as control (**c**); only very rare dead cells stained in blue (arrows) are present in all the treatments. (**d**) Leaf disk from a non-treated leaf inoculated with 40 µg/mL of TNV, at 72 h after infection: virus lesions formed by numerous dead cells (in dark blue) surrounded by less damaged cells (arrows) are expanding in the tissue; an enlargement of the framed part of a lesion is visible in the inset. (**e**) Leaf inoculated with a mixture of 2 µg/mL PD-L4 and 40 µg/mL TNV (1:1), showing only small groups of damaged cells, enlarged in the inset (arrows); (**f**) Leaf treated with 2 µg/mL PD-L4 in the abaxial surface and inoculated soon after with 40 µg/mL of TNV in the adaxial surface: a TNV lesion is developing at 72 h after infection; the lesion is formed by a few dead cells (in dark blue) and numerous damaged cells around (arrows) and is still invisible at naked eyes. All bars = 200 µm.

To shed further light on the antiviral activity of these RIPs, bean leaves only treated with PD-L1 or PD-L4 at 2 µg/mL or 10 µg/mL were stained with Evans blue for the detection of cell death at 24, 48, and 72 h after treatment. Dead cells were detected only occasionally after PD-L1 and PD-L4 treatments at both concentrations and at any tested time point (Figure 5a–c) suggesting that these RIPs either are not able to permeate into cells or, once entered, they do not cause enough damages to induce cell death, for at least 72 h (Figure 5a–c). Evans blue staining was also performed on leaves only inoculated with TNV or simultaneously inoculated with PD-L4 (2 µg/mL) and TNV, at 72 h after inoculation on the onset of virus lesion appearance. In leaves inoculated with TNV only the developing lesions were clearly visible as groups of dead cells, surrounded by less damage cells (Figure 5d). These lesions matured in the following two days in typical necrotic lesions clearly visible at naked eyes. Instead, in leaves inoculated simultaneously with a mixture of PD-L4 and TNV (1:1) lesions were very small and formed by a few damaged cells (Figure 5e). Only rare of such damaged cell groups enlarged in the following two days becoming visible necrotic lesions. Finally, in leaves inoculated with TNV (adaxially) and PD-L4 applied abaxially virus lesions were formed by a discrete number of damaged cells, though only few of them appeared dark blue and thus certainly died (Figure 5f). In the following the days, some of these lesions matured in visible necrotic lesions.

**Figure 6:**
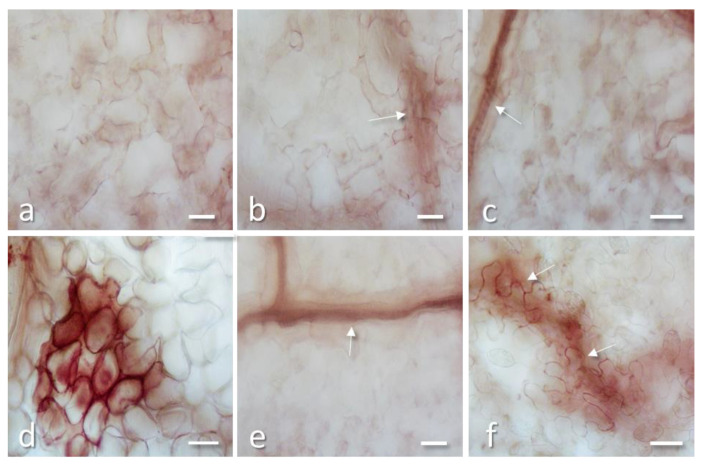
3,3′-Diaminobenzidine (DAB) staining of bean leaf disks 48 h after treatment with PD-L1 2 µg/mL (**a**) or PD-L4 2 µg/mL (**b**) or water as control (**c**); no staining is present in the tissues, except for veins (arrows) that are stained in brown as they contain H_2_O_2_ for cell wall lignification. (**d**) Leaf disk from a non-treated leaf inoculated with 40 µg/mL of TNV, at 48 h after infection: a developing virus lesion, densely stained for the presence of H_2_O_2_ due to the oxidative stress induced by the virus, is shown. (**e**) Leaf inoculated with a mixture of 2 µg/mL PD-L4 and 40 µg/mL TNV (1:1), no staining is present except for the veins (arrow). (**f**) Leaf treated with 2 µg/mL PD-L4 in the abaxial surface and inoculated soon after with 40 µg/mL of TNV in the adaxial surface: some DAB staining is present at 48 h in walls of cells possibly involved in an early formation of a small lesion (arrows), as the one showed in Figure 5f. All bars = 50 µm.

3,3′-Diaminobenzidine (DAB) staining of bean leaf disks 48 h after treatment with PD-L1 2 µg/mL or PD-L4 2 µg/mL showed that no H_2_O_2_ deposits were present in the tissues, except for veins that were stained in brown because they normally contain H_2_O_2_ for cell wall lignification (Figure 6a–c). This suggest that these RIPs are not able to induce oxidative burst, either because they do not possess this property or are unable to permeate into intact cells. A strong H_2_O_2_ deposition was instead elicited by TNV infection due to the hypersensitive reaction (HR) caused by the virus on the onset of infection, when lesions were not yet visible at naked eyes (Figure 6d).

Leaf inoculated simultaneously with a mixture of RIP and TNV showed no DAB staining except for the veins (Figure 6e; arrow), indicating that virus inhibition occurs at a very early stage of infection. Finally, leaf treated with RIP in the abaxial surface and inoculated soon after with TNV in the adaxial surface showed after 48 h some DAB staining in walls of cells possibly involved in an early formation of a small lesion (Figure 6f).

The above microscopic observations confirm that the strongest antiviral activity is exerted when virus and RIP are inoculated together into the leaves, reducing almost completely cell damages. Considering that RIPs should not have access to viral RNA in assembled virus particles [43], it is likely that the antiviral activity is mainly exerted directly into the cells when the virus disassembles, possibly damaging both viral and ribosome RNA, therefore impairing virus replication. In this context, it seems determinant that both virus and RIPs enter the cells at the same time. In fact, by inoculating the virus in the adaxial leaf surface and applying PD-L4 in the abaxial surface, there could be a delay in RIP permeation into the infected cells. This would allow the virus to replicate itself, until it is blocked by the RIP, as suggested by the discrete lesions visible in Figure 5f and by H_2_O_2_ deposition in Figure 6f.

## 3. Conclusions

Type-1 RIPs from *P. dioica* are extensively characterized from a structural and enzymatic point of view (for a summary see [30]). Vice versa, few information is known about their biological action that could justify the presence of these enzymes in the various organs of *P. dioica*. On the other hand, recent studies have shown that type-1 RIPs from *P. dioica* display several biological and antipathogenic activities being adept at damaging the tobacco mosaic virus RNA and to inhibit the growth of *Penicillium digitatum* [6]. Thus in this work, we wanted to test the antiviral activity of both PD-L1 and PD-L4, the most expressed type-1 RIPs in the leaves of *P. dioica*, against TNV by using as a host *P. vulgaris*, cv. Borlotto Nano Lingua di Fuoco (BLF).

Data show that both toxins have the ability to reduce the number of lesions when applied on *P. vulgaris* leaves in the same leaf surface compared to leaves inoculated with the virus alone. In particular, the decrease in lesions is particularly evident in the presence of PD-L4 with respect to PD-L1. The different action of the two toxins could justify their different expression, considering that PD-L4 is more expressed in the leaves growing during spring and summer and in minor amount in the autumn and winter [35]. On the other hand, PD-L1 expressed in autumn and winter [35] shows a minor antiviral activity that could be implicated in different physiological roles such as leaf senescence [44].

Moreover, a decrease in antiviral activity for PD-L4 occurred when the virus suspension was inoculated either on the adaxial or abaxial leaf surface and PD-L4 on the opposite leaf surface.

Therefore, it is likely that PD-Ls antiviral activity is fully expressed only when both RIPs and virus are present in the same cells, whether they had entered together or separately. At this regard, the absence of dead cells in leaves only treated with PD-L1 and PD-L4 suggests that these proteins cannot be internalized, at least in a sufficient amount to cause cell death. It is more likely that they enter easily in those cells already damaged by the virus, thus accelerating their death and hampering virus spreading. Whether these RIPs, once present in the virus infected cells, inhibit viral RNA translation and replication by depurination of host ribosomal RNA, or directly depurinate viral RNA, as recently demonstrate for PAP [45], it is not known. Possibly there is a synergistic effect of these activities, also taking into account that in vitro PD-L4 degradation capacity against tobacco mosaic virus (TMV) RNA has previously been shown to be only partially effective [6].

## 4. Material and Methods

### 4.1. Materials

Materials for chromatography were described elsewhere [28,46]. All other reagents and chemicals were of analytical grade (Sigma-Aldrich/Merck Life Science S.r.l., Milano, Italy).

### 4.2. Plant Materials for Type-1 RIP Purifications and for Antiviral Assays

Type-1 RIPs, namely PD-L1 and PD-L4 were from fully expanded leaves, collected from a single female adult tree plant of *Phytolacca dioica*, growing in the Botanical Garden of the University of Naples “Federico II” (Italy). *Phaseolus vulgaris* plants, cv. Borlotto Nano Lingua di Fuoco (BLF) were grown in a greenhouse at 24 ± 2 °C, RH 60 ± 5%, 16 h/8 h light/dark period and used when primary leaves were completely expanded.

### 4.3. Protein Purification

PD-L1 and PD-L4 were purified according to the procedure previously reported [33]. Briefly, the raw extract was acidified with acetic acid and subjected to consecutive chromatographic steps: Streamline™ SP (GE Healthcare, Milano, Italy) step wise; gel-filtration by Sephadex G-75 Hi-load 26/60 column (GE Healthcare) on an Akta purification system. Finally, a final low-pressure cation exchange chromatography step on a CM-52 column (GE Healthcare Whatman, Chicago, Il, USA) eluted with a NaCl gradient. Fractions corresponding to main peaks (PD-Ls) with activity inhibitory to cell-free protein synthesis were checked by SDS-PAGE analysis, pooled dialyzed against water, freeze-dried and stored at −20 °C until use.

### 4.4. Biochemical Analytical Procedures

General methodology using for analytical biochemical characterization (SDS-PAGE and protein concentration by bicinchoninic acid (BCA) assay) are reported in detail in previously publisher paper [46]. As molecular markers for SDS-PAGE, SigmaMarker™ (Sigma-Aldrich, St. Louis, Missouri, USA) low range, mol wt 6500–66,000 Da (product code M3913) were used.

### 4.5. Homogeneity of Protein by Capillary Electrophoresis

Capillary Electrophoresis (CE) in sodium dodecyl sulphate (SDS) was carried out on a Beckman P/ACE System 5550, using the eCAP^TM^ SDS 14–200 kit in a 47 cm capillary, monitoring at 214 nm following manufacturer’s instructions (Beckman Coulter SRL, Cassina de’Pecchi (Milano), Italy).

### 4.6. Tobacco Necrosis Virus (TNV) Purification

Symptomatic *Nicotiana benthamiana* plants, previously inoculated with a TNV-D strain gently supplied by Institute for Sustainable Plant Protection (IPSP), Turin, Italy, were used for virus purification. Frozen material was ground in cold 0.1 M ammonium citrate buffer (1:3 *w*/*v*), filtered, clarified by differential centrifugation as described by [47] and further purified by ultracentrifugation through sucrose density gradient (10–40% in distilled water), at 150,000× *g* for 40 min. A light scattering virus band was recovered from the gradients and concentrated by centrifugation at 150,000× *g* for 4 h. Finally, virus sediment was resuspended in distilled water at the final concentration of 40 µg/mL and stored at −80 °C until utilization.

### 4.7. PD-Ls Treatments and Virus Inoculation

Plants were inoculated on the first developed leaf by rubbing with 1 mL/leaf mixture of PD-L1 or PD-L4 (2 µg/mL or 10 µg/mL) and purified virus (1:1), using a 600 mesh carborundum as an abrasive. The final virus concentration in the inoculum was of 100 ng/mL. In some other experiments, PD-L4 and virus were inoculated separately on the abaxial and adaxial surface of the same leaf. As controls, some plants were inoculated respectively with: (1) PD-L1; (2) PD-L4; (3) TNV; (4) water.

### 4.8. Evaluation of Antiviral Activity

Plants were observed for the development of lesions for 4–5 days. When lesions were fully developed, infected leaves were detached and immediately scanned at 300 DPI resolution. Images were analyzed with Global Lab (Data Translation, Marlborough, MA, USA) to determine the number of lesions and PD-Ls inhibitory activity was calculated as percent reduction of this number in respect to controls.

### 4.9. Histo-Cytochemistry

Five leaf disks, 1 cm in diameter, were randomly punched with a cork-bore from PD-Ls or TNV or PD-Ls + TNV inoculated bean leaves at two days post-inoculation (dpi) and stained with Evans blue, to identify dead cell in the tissues and with 3,3′- Diaminobenzidine (DAB) to localize H_2_O_2_ deposits, following a previously reported protocol [48]. Samples were examined with an Olympus B×50 light microscope (Olympus, Shinjuku, Tokyo, Japan), equipped with differential interference contrast (DIC) and epi-polarization filters.

### 4.10. Statistical Analysis

For the antiviral assay on bean plants, results are expressed as mean ± standard deviation of data collected from at least three independent experiments, with 5 bean plants and 10 treated/inoculated leaves for each treatment. Data were subjected to one-way analysis of variance, and comparison among means was determined according to Fisher’s least significant difference test. Significant differences were determined at *p* < 0.05.

## Figures and Tables

**Table 1:** Inhibitory effect of PD-L1 and PD-L4, expressed as percent reduction of virus lesions in comparison with control plants inoculated with the virus only. Values are means (± SD) of triplicate analyses (*n* = 3).

Inoculation Method	% Reduction of TNV-Lesions
TNV + PD-L1 2 µg/mL on the same leaf surface	89.1 ± 3
TNV + PD-L1 10 µg/mL on the same leaf surface	95.8 ± 2
TNV + PD-L4 2 µg/mL on the same leaf surface	98.7 ± 4
TNV + PD-L4 10 µg/mL on the same leaf surface	99.7 ± 2
TNV on the adaxial leaf surface and PD-L4 on the abaxial	72.6 ± 3
TNV on the abaxial leaf surface and PD-L4 on the adaxial	78.0 ± 3

## References

[1] Stirpe F. (2013). Ribosome-inactivating proteins: From toxins to useful proteins. Toxicon.

[2] Endo Y., Tsurugi K. (1987). RNA *N*-glycosidase activity of ricin A-chain. Mechanism of action of the toxic lectin ricin on eukaryotic ribosomes. J. Biol. Chem..

[3] Brigotti M., Rambelli F., Zamboni M., Montanaro L., Sperti S. (1989). Effect of alpha-sarcin and ribosome-inactivating proteins on the interaction of elongation factors with ribosomes. Biochem. J..

[4] Bolognesi A., Polito L., Lubelli C., Barbieri L., Parente A., Stirpe F. (2002). Ribosome-inactivating and adenine polynucleotide glycosylase activities in *Mirabilis jalapa* L. tissues. J. Biol. Chem..

[5] Aceto S., Di Maro A., Conforto B., Siniscalco G.G., Parente A., Delli Bovi P., Gaudio L. (2005). Nicking activity on pBR322 DNA of ribosome inactivating proteins from *Phytolacca dioica* L. leaves. Biol. Chem..

[6] Iglesias R., Citores L., Ragucci S., Russo R., Di Maro A., Ferreras J.M. (2016). Biological and antipathogenic activities of ribosome-inactivating proteins from *Phytolacca dioica* L. Biochim. Biophys. Acta Gen. Subj..

[7] Barbieri L., Battelli M.G., Stirpe F. (1993). Ribosome-inactivating proteins from plants. Biochim. Biophys. Acta.

[8] De Zaeytijd J., Van Damme E.J. (2017). Extensive evolution of cereal ribosome-inactivating proteins translates into unique structural features, activation mechanisms, and physiological roles. Toxins.

[9] Peumans W.J., Shang C., Van Damme E.J.M., Stirpe F., Lappi D.A. (2014). Updated model of the molecular evolution of RIP genes. Ribosome-Inactivating Proteins.

[10] Stirpe F., Gilabert-Oriol R., Carlini C.R., Ligabue-Braun R. (2017). Ribosome-inactivating proteins: An overview. Plant Toxins.

[11] Ferreras J.M., Citores L., Iglesias R., Jiménez P., Girbés T., Lord J.M., Hartley M.R. (2010). Sambucus ribosome-inactivating proteins and lectins. Toxic Plant Proteins.

[12] Hey T.D., Hartley M., Walsh T.A. (1995). Maize ribosome-inactivating protein (b-32). Homologs in related species, effects on maize ribosomes, and modulation of activity by pro-peptide deletions. Plant Physiol..

[13] Stirpe F. (2004). Ribosome-inactivating proteins. Toxicon.

[14] Zeng M., Zheng M., Lu D., Wang J., Jiang W., Sha O. (2015). Anti-tumor activities and apoptotic mechanism of ribosome-inactivating proteins. Chin. J. Cancer.

[15] Vago R., Marsden C.J., Lord J.M., Ippoliti R., Flavell D.J., Flavell S.-U., Ceriotti A., Fabbrini M.S. (2005). Saporin and ricin A chain follow different intracellular routes to enter the cytosol of intoxicated cells. FEBS J..

[16] de Virgilio M., Lombardi A., Caliandro R., Fabbrini M.S. (2010). Ribosome-inactivating proteins: From plant defense to tumor attack. Toxins.

[17] Pizzo E., Di Maro A. (2016). A new age for biomedical applications of Ribosome Inactivating Proteins (RIPs): From bioconjugate to nanoconstructs. J. Biomed. Sci..

[18] Rust A., Partridge L.J., Davletov B., Hautbergue G.M. (2017). The use of plant-derived ribosome inactivating proteins in immunotoxin development: Past, present and future generations. Toxins.

[19] Polito L., Djemil A., Bortolotti M. (2016). Plant toxin-based immunotoxins for cancer therapy: A short overview. Biomedicines.

[20] Zhu F., Zhou Y.-K., Ji Z.-L., Chen X.-R. (2018). The plant ribosome-inactivating proteins play important roles in defense against pathogens and insect pest attacks. Front. Plant Sci..

[21] Fabbrini M.S., Katayama M., Nakase I., Vago R. (2017). Plant ribosome-inactivating proteins: Progesses, challenges and biotechnological applications (and a few digressions). Toxins.

[22] Di Maro A., Citores L., Russo R., Iglesias R., Ferreras J.M. (2014). Sequence comparison and phylogenetic analysis by the Maximum Likelihood method of ribosome-inactivating proteins from angiosperms. Plant Mol. Biol..

[23] Lapadula W.J., Ayub M.J. (2017). Ribosome inactivating proteins from an evolutionary perspective. Toxicon.

[24] Parente A., Chambery A., Di Maro A., Russo R., Severino V., Stirpe F., Lappi D.A. (2014). Ribosome-inactivating proteins from Phytolaccaceae. Ribosome-Inactivating Proteins.

[25] Domashevskiy A.V., Goss D.J. (2015). Pokeweed antiviral protein, a ribosome inactivating protein: Activity, inhibition and prospects. Toxins.

[26] Koch P.E., Bonness M.S., Lu H., Mabry T.J. (1996). Protoplasts from *Phytolacca dodecandra* L’Herit (endod) and *P. americana* L. (pokeweed). Plant Cell Rep..

[27] Corrado G., Bovi P.D., Ciliento R., Gaudio L., Di Maro A., Aceto S., Lorito M., Rao R. (2005). Inducible expression of a *Phytolacca heterotepala* ribosome-inactivating protein leads to enhanced resistance against major fungal pathogens in tobacco. Phytopathology.

[28] Di Maro A., Chambery A., Daniele A., Casoria P., Parente A. (2007). Isolation and characterization of heterotepalins, type 1 ribosome-inactivating proteins from *Phytolacca heterotepala* leaves. Phytochemistry.

[29] Moon Y.H., Song S.K., Choi K.W., Lee J.S. (1997). Expression of a cDNA encoding *Phytolacca insularis* antiviral protein confers virus resistance on transgenic potato plants. Mol. Cells.

[30] Parente A., Berisio R., Chambery A., Di Maro A., Lord J.M., Hartley M.R. (2010). Type 1 ribosome-Inactivating Proteins from the ombú tree (*Phytolacca dioica* L.). Toxic Plant Proteins.

[31] Chambery A., Di Maro A., Parente A. (2008). Primary structure and glycan moiety characterization of PD-Ss, type 1 ribosome-inactivating proteins from *Phytolacca dioica* L. seeds, by precursor ion discovery on a Q-TOF mass spectrometer. Phytochemistry.

[32] Di Maro A., Berisio R., Ruggiero A., Tamburino R., Severino V., Zacchia E., Parente A. (2012). Structural and enzymatic properties of an in vivo proteolytic form of PD-S2, type 1 ribosome-inactivating protein from seeds of *Phytolacca dioica* L. Biochem. Biophys. Res. Commun..

[33] Di Maro A., Valbonesi P., Bolognesi A., Stirpe F., De Luca P., Siniscalco Gigliano G., Gaudio L., Delli Bovi P., Ferranti P., Malorni A. (1999). Isolation and characterization of four type-1 ribosome-inactivating proteins, with polynucleotide:adenosine glycosidase activity, from leaves of *Phytolacca dioica* L. Planta.

[34] Di Maro A., Chambery A., Carafa V., Costantini S., Colonna G., Parente A. (2009). Structural characterization and comparative modeling of PD-Ls 1-3, type 1 ribosome-inactivating proteins from summer leaves of *Phytolacca dioica* L. Biochimie.

[35] Parente A., Conforto B., Di Maro A., Chambery A., De Luca P., Bolognesi A., Iriti M., Faoro F. (2008). Type 1 ribosome-inactivating proteins from *Phytolacca dioica* L. leaves: Differential seasonal and age expression, and cellular localization. Planta.

[36] Russo R., Chambery A., Severino V., Parente A., Di Maro A. (2015). Structural characterization of dioicin 1 from *Phytolacca dioica* L. gains novel insights into phylogenetic relationships of Phytolaccaceae type 1 RIPs. Biochem. Biophys. Res. Commun..

[37] Faoro F., Conforto B., Di Maro A., Parente A., Iriti M. Activation of plant defence response contributes to the antiviral activity of Diocin 2 from *Phytolacca dioica*. Proceedings of the IOBC/WPRS Working Group “Induced Resistance in Plants Against Insects and Diseases”.

[38] Pizzo E., Zanfardino A., Di Giuseppe A.M.A., Bosso A., Landi N., Ragucci S., Varcamonti M., Notomista E., Di Maro A. (2015). A new active antimicrobial peptide from PD-L4, a type 1 ribosome inactivating protein of *Phytolacca dioica* L.: A new function of RIPs for plant defence?. FEBS Lett..

[39] Pizzo E., Pane K., Bosso A., Landi N., Ragucci S., Russo R., Gaglione R., Torres M.D.T., de la Fuente-Nunez C., Arciello A. (2018). Novel bioactive peptides from PD-L1/2, a type 1 ribosome inactivating protein from *Phytolacca dioica* L. Evaluation of their antimicrobial properties and anti-biofilm activities. Biochim. Biophys. Acta Biomembr..

[40] Rubino L., Matelli G.P., Mahy B.W.J., van Regenmortel M.H.V. (2010). Necrovirus. Desk Encyclopedia of Plant and Fungal Virology.

[41] Iriti M., Faoro F. (2008). Abscisic acid is involved in chitosan-induced resistance to tobacco necrosis virus (TNV). Plant Physiol. Biochem..

[42] Iriti M., Sironi M., Gomarasca S., Casazza A.P., Soave C., Faoro F. (2006). Cell death-mediated antiviral effect of chitosan in tobacco. Plant Physiol. Biochem..

[43] Battelli M.G., Stirpe F., Chessin M., DeBorde D., Zipf A. (1995). Ribosome-inactivating poteins from plants. Antiviral Proteins in Higher Plants.

[44] Stirpe F., Barbieri L., Gorini P., Valbonesi P., Bolognesi A., Polito L. (1996). Activities associated with the presence of ribosome-inactivating proteins increase in senescent and stressed leaves. FEBS Lett..

[45] Picard D., Kao C.C., Hudak K.A. (2005). Pokeweed antiviral protein inhibits brome mosaic virus replication in plant cells. J. Biol. Chem..

[46] Landi N., Pacifico S., Ragucci S., Iglesias R., Piccolella S., Amici A., Di Giuseppe A.M.A., Di Maro A. (2017). Purification, characterization and cytotoxicity assessment of Ageritin: The first ribotoxin from the basidiomycete mushroom *Agrocybe aegerita*. Biochim. Biophys. Acta Gen. Subj..

[47] Zhang L., French R., Langenberg W.G. (1993). Molecular cloning and sequencing of the coat protein gene of a Nebraskan isolate of tobacco necrosis virus: The deduced coat protein sequence has only moderate homology with those of strain A and strain D. Arch. Virol..

[48] Faoro F., Iriti M. (2005). Cell death behind invisible symptoms: Early diagnosis of ozone injury. Biol. Plant..

